# A Two-Stage Culture Strategy for *Scenedesmus* sp. FSP3 for CO_2_ Fixation and the Simultaneous Production of Lutein under Light and Salt Stress

**DOI:** 10.3390/molecules27217497

**Published:** 2022-11-03

**Authors:** Jiawei Li, Xinqing Zhao, Jo-Shu Chang, Xiaoling Miao

**Affiliations:** 1 State Key Laboratory of Microbial Metabolism, School of Life Sciences & Biotechnology, Shanghai Jiao Tong University, 800 Dongchuan Road, Shanghai 200240, China; 2 Joint International Research Laboratory of Metabolic & Developmental Sciences, Shanghai Jiao Tong University, Shanghai 200240, China; 3 Biomass Energy Research Center, Shanghai Jiao Tong University, Shanghai 200240, China; 4 Department of Chemical and Materials Engineering, Tunghai University, Taichung 407, Taiwan; 5 Research Center for Smart Sustainable Circular Economy, Tunghai University, Taichung 407, Taiwan; 6 Department of Chemical Engineering, National Cheng-Kung University, Tainan 701, Taiwan; 7 Department of Chemical Engineering and Materials Science, Yuan Ze University, Taoyuan 320, Taiwan

**Keywords:** lutein, *Scenedesmus* sp. FSP3, CO_2_ fixation, light stress, salt stress

## Abstract

In this study, *Scenedesmus* sp. FSP3 was cultured using a two-stage culture strategy for CO_2_ fixation and lutein production. During the first stage, propylene carbonate was added to the medium, with 5% CO_2_ introduced to promote the rapid growth and CO_2_ fixation of the microalgae. During the second stage of cultivation, a NaCl concentration of 156 mmol L^−1^ and a light intensity of 160 μmol m^−2^ s^−1^ were used to stimulate the accumulation of lutein in the microalgal cells. By using this culture method, high lutein production and CO_2_ fixation were simultaneously achieved. The biomass productivity and carbon fixation rate of *Scenedesmus* sp. FSP3 reached 0.58 g L^−1^ d^−1^ and 1.09 g L^−1^ d^−1^, with a lutein content and yield as high as 6.45 mg g^−1^ and 2.30 mg L^−1^ d^−1^, respectively. The results reveal a commercially feasible way to integrate microalgal lutein production with CO_2_ fixation processes.

## 1. Introduction

Since the 21st century, global carbon emissions have accelerated sharply, with global CO_2_ emissions increasing by 40% from 2000–2019. In addition, global CO_2_ emissions rebounded in 2021, reaching their highest-ever annual level of 36.3 Gt, according to the International Energy Agency (IEA) [1]. The level of atmospheric CO_2_ concentration reached 419 ppm in May 2021, which is the highest on record and continues to increase. It will further cause serious environmental problems, such as the greenhouse effect, global warming, ocean acidification, etc. affecting the sustainable development of human society and people’s life and health.

CO_2_ fixation based on microalgae is considered one of the most promising and sustainable carbon capture, utilization, and storage (CCUS) technologies [2]. Microalgae are microorganisms with chloroplasts that are capable of photosynthesis. Marine phytoplankton represented by microalgae are responsible for 40% of global CO_2_ fixation each year. Microalgae have a larger specific surface area and are more efficient at chloroplast photosynthesis compared to other higher plant cells. Moreover, their own carbon concentration mechanism (CCM), meaning their carbon sequestration rate, is much higher than that of terrestrial plants, which has great potential for development [3].

Microalgae can efficiently use light energy, CO_2_, and water for photosynthesis to produce oxygen and synthesize carbohydrates that store energy. Through further biochemical reactions, various nutrients such as proteins and oils can be synthesized, and many unique biologically active substances can also be synthesized, such as unsaturated fatty acids (Docosahexaenoic acid (DHA), Eicosapentaenoic acid (EPA), etc.), carotenoids (β-carotene, astaxanthin, lutein, zeaxanthin, etc.), pigment-protein complexes (phycocyanin, phycoerythrin, etc.), polysaccharides, active polypeptides, and other high-value-added products [4]. Obtaining bioactive substances from microalgae has become a hot topic in the development and utilization of microalgal resources.

Lutein is a photoprotective carotenoid, which is closely related to the light capture complex (LHC) of photosynthetic devices. It can deactivate triplet chlorophyll (3Chl^⁎^) and remove excess ROS (reactive oxygen species). Furthermore, lutein is involved in the nonphotochemical quenching (NPQ) of excess light energy in PSII antennas [5]. Therefore, lutein accumulates to protect photosynthetic devices during stress caused by the environment or changes in light intensity. Lutein is also related to human health, and the usefulness of lutein has recently been widely recognized. It has an excellent antioxidant effect, vision protection effect, anticancer effect, and food-coloring effect. It is widely used in medicine, health food, cosmetics, and the feed industry [6]. Therefore, the market price of natural lutein has exceeded EUR 10 million t^−1^ [7]. The traditional way to obtain natural lutein is to extract it from marigold. However, the lutein content (0.3 mg g^−1^) in marigold is extremely low, and the bioavailability is extremely poor. Microalgae have a higher growth rate and lutein content compared to marigolds. The biochemical profiles of microalgae exhibit great metabolic plasticity and can adapt to changes in environmental conditions. When facing different environmental stresses, the content of microalgae lutein will accumulate. In recent years, microalgae have proven to be the most promising source of lutein [8].

However, the cost of lutein production based on microalgae is still expensive, and commercial production is still not available, so the economic benefits need to be further improved. On the one hand, the economic benefits can be improved by enhancing microalgae lutein production, and the lutein production process of microalgae can be integrated into the CO_2_ fixation process to obtain additional benefits. This study aims to address these two issues; a two-stage strategy was adopted to cultivate microalgae to achieve lutein production and CO_2_ fixation simultaneously.

## 2. Results

### 2.1. Growth and CO_2_ Fixation Rates of Microalgae under Different CO_2_ Concentrations

**Figure 1:**
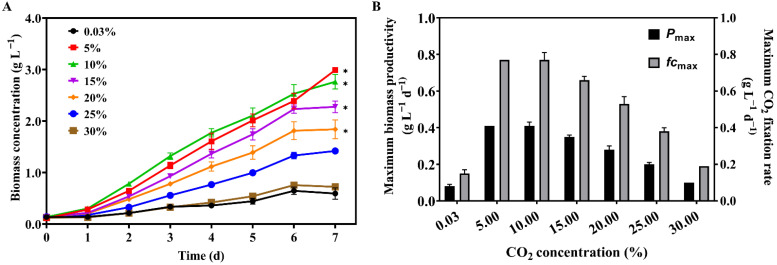
Growth (**A**) and the maximum biomass productivity (*P*max) and maximum CO_2_ fixation rate (*fc*max) (**B**) of *Scenedesmus* sp. FSP3 under different CO_2_ concentrations. Dunnett’s test was performed to determine statistically significant difference: *, *p* < 0.05, n = 3.

In general, CO_2_ concentrations below 5% are suitable for microalgal growth [9], while higher CO_2_ concentrations are considered harmful to microalgal growth. Our results show that *Scenedesmus* sp. FSP3 had a high tolerance to CO_2_, which can grow well under 30% CO_2_ (Figure 1A). As is shown in Figure 1A, *Scenedesmus* sp. FSP3 showed higher biomass concentration, biomass productivity, and CO_2_ fixation rate under CO_2_ concentrations ranging from 5 to 30% when compared to that under 0.03%. Notably, the growth of *Scenedesmus* sp. FSP3 greatly enhanced at a CO_2_ concentration of 5%, reaching a maximum biomass concentration of 2.99 g L^−1^ after seven days of cultivation (Figure 1A). The maximum biomass productivity and CO_2_ fixation rate of *Scenedesmus* sp. FSP3, under a CO_2_ concentration of 5%, was 0.41 g L^−1^ d^−1^ and 0.77 g L^−1^ d^−1^ (Figure 1B), respectively. Therefore, a 5% CO_2_ concentration was selected for further study.

### 2.2. Addition of CO_2_ Solubilization Enhancer for Better Carbon Absorption

The solubility of CO_2_ in the culture solution has a great influence on the growth of microalgae [10]. However, the solubility of CO_2_ in water is only 1.45 g L^−1^ at 25 °C and 1 atm, so when CO_2_ is introduced into the culture medium, the short residence time and slow diffusion of CO_2_ in the culture causes most of the CO_2_ to escape [11], which limits the carbon sequestration of the microalgae in the medium. The addition of a CO_2_ solubilization enhancer was reported to be effective in promoting the solubility of CO_2_ and carbon sequestration of microalgae [10,11,12].

In our study, a 5% CO_2_ concentration was used for the cultivation, and methanol, polyethylene glycol (PEG200), propylene carbonate (PC), polyethylene glycol dimethyl ether (NHD), and N-methylpyrrolidone (NMP) were selected as the CO_2_ solubilization enhancers. They were reported to be harmless to microalgae within the range of concentrations used in our present study [11], except for PEG200, which is not metabolized by microalgae.

**Figure 2:**
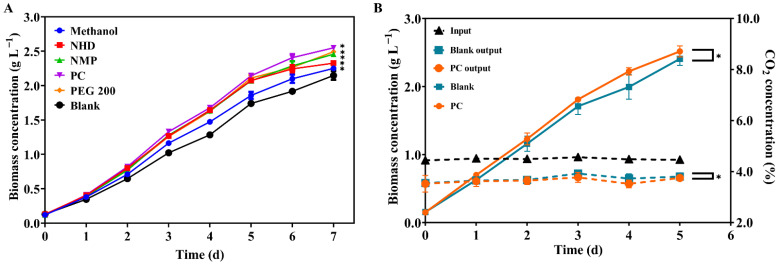
Growth of *Scenedesmus* sp. FSP3 with different CO_2_ solubilization enhancers under 5% CO_2_ (**A**). NHD: polyethylene glycol dimethyl ether, NMP: N-methylpyrrolidone, PC: propylene carbonate, PEG200: polyethylene glycol 200. (**B**) The input and output CO_2_ concentrations of the microalgae cultures (the dotted line) and the growth (the solid line) of *Scenedesmus* sp. FSP3 with PC added under 5% CO_2._ (Input: the concentration of CO_2_ into the medium with PC added and blank; PC output: the effluent CO_2_ concentration of the medium with PC added; Blank output: The effluent CO_2_ concentration of blank; PC: the growth of *Scenedesmus* sp. FSP3 in the medium with PC added; Blank: the growth of *Scenedesmus* sp. FSP3 in the blank. Dunnett’s test was performed to determine statistically significant difference: *, *p* < 0.05, n = 3.

As is shown in Figure 2A, after seven days of cultivation, the biomass concentrations were 2.25, 2.32, 2.46, 2.55, and 2.49 g L^−1^ in those cultures supplemented with 5 mmol L^−1^ methanol, NHD, NMP, PC, and PEG200, respectively. In comparison, the biomass concentration obtained in the blank was 2.12 g L^−1^. The growth of the microalgae was promoted to varying degrees under all conditions, while the addition of PC achieved the most obvious growth promotion effect, with its biomass concentration increased by 20.28% when compared to the blank.

The effect of PC on CO_2_ absorption was further investigated, and the results suggested that the growth and CO_2_ absorption significantly improved under the addition of PC compared with the blank. As is shown in Figure 2B, the concentration of CO_2_ in the medium (Input) with PC added or as a blank was basically constant, with an average concentration of 4.49%. In general, the CO_2_ absorption rate of the medium with PC and the blank was 18.98% and 17.14%, respectively. The effluent CO_2_ concentration of the medium with the PC added (PC output) and the effluent CO_2_ concentration of the blank (Blank output) showed little difference during the first two days; as the biomass concentration gap between the PC added and the blank increased in the subsequent cultures, the PC output showed a lower CO_2_ concentration (Figure 2B). The culture medium with added PC absorbed more CO_2_ and had a lower CO_2_ concentration output. On the fourth day, the difference in the CO_2_ absorption rate for the PC added and the blank reached a maximum of 4.69%, along with the largest biomass concentration difference (Figure 2B). Maximum biomass productivity reached 0.55 g L^−1^ d^−1^; meanwhile the CO_2_ fixation rate reached 1.03 g L^−1^ d^−1^ under a 5% CO_2_ concentration with added PC.

### 2.3. Two-Stage Strategy Promoting Lutein Accumulation under Light and Salt Stress

*Scenedesmus* sp. FSP3 were cultured using a two-stage culture strategy. In the first stage, *Scenedesmus* sp. FSP3 were cultivated under a light intensity of 90 μmol m^−2^ s^−1^ and 5% CO_2_ infusion to facilitate cell growth and CO_2_ fixation. Furthermore, in the second stage, the microalgae were cultivated under stress to stimulate lutein accumulation. In previous reports, the lutein levels were influenced by temperature, light intensity, salt concentration, nitrogen source availability, oxidation, and other factors [13]. According to the preliminary experiment, we investigated the effects of light stress and salt stress on the lutein content of *Scenedesmus* sp. FSP3 in the second stage culture.

#### 2.3.1. Effect of Light Intensity on Lutein Content

**Figure 3:**
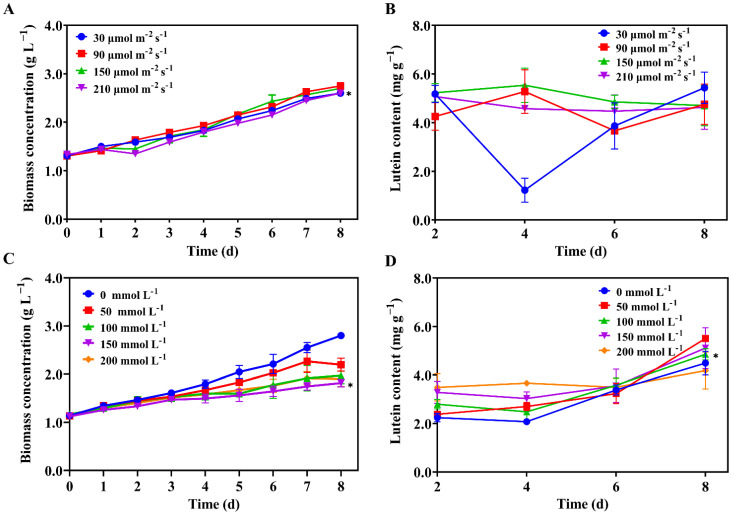
Effect of different light intensities and NaCl concentrations on the growth (**A**,**C**) and lutein content (**B**,**D**) of *Scenedesmus* sp. FSP3 in the second stage, (The 90 μmol m^−2^ s^−1^ light intensity used in the NaCl experiments. Dunnett’s test was performed to determine statistically significant difference: *, *p* < 0.05, n = 3).

In the present study, different light intensities of 30, 90, 150, and 210 μmol m^−2^ s^−1^ were set to investigate the growth and dynamics of the lutein content of *Scenedesmus* sp. FSP3. As is shown in Figure 3A, *Scenedesmus* sp. FSP3 showed little difference in growth under these four light intensities, and the highest biomass concentration (2.75 g L^−1^ d^−1^) was obtained at 90 μmol m^−2^ s^−1^ (Figure 3A).

The lutein content under a light intensity of 30 μmol m^−2^ s^−1^ was the highest on the eighth day, reaching 5.44 mg g^−1^. Under a 90 and 210 μmol m^−2^ s^−1^ light intensity, *Scenedesmus* sp. FSP3 showed a low accumulation of lutein content, and the peak lutein content was achieved on day four, at 150 μmol m^−2^ s^−1^ with 5.54 mg g^−1^ in our study (Figure 3B).

#### 2.3.2. Effect of Salt Concentration on Lutein Content

According to the preliminary experiment, NaCl concentration gradients of 0, 50, 150, and 200 mmol L^−1^ were set to compare the dynamic changes in the growth and lutein content of *Scenedesmus* sp. FSP3 at different NaCl concentrations. As is shown in Figure 3C, *Scenedesmus* sp. FSP3 grew best in the medium with a 0 mmol L^−1^ NaCl concentration, while the growth of *Scenedesmus* sp. FSP3 was inhibited in the medium with a 50–200 mmol L^−1^ NaCl concentration, with little difference.

During the period from four to eight days, the lutein content of those microalgae under a 0–150 mmol L^−1^ salt concentration increased with the time of stress, reaching a maximum of 5.51 mg g^−1^ under a 50 mmol L^−1^ NaCl concentration on day 8 (Figure 3D). On the eighth day, the lowest lutein content of *Scenedesmus* sp. FSP3 was found under a 200 mmol L^−1^ NaCl concentration. This is probably because the high salt concentration caused damage to the microalga cells, resulting in a reduction in lutein content. Therefore, the appropriate NaCl concentration is also crucial for the accumulation of lutein in microalgae.

### 2.4. Response Surface Methodology for Lutein Production

**Figure 4:**
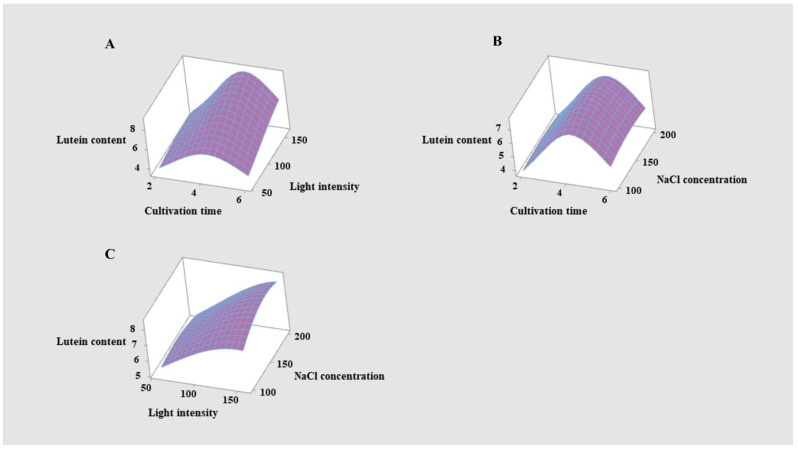
Response surface of lutein content. (**A**) Response surface for different light intensities and cultivation times on lutein content. (**B**) Response surface for different NaCl concentrations and cultivation times on lutein content. (**C**) Response surface for different light intensities and NaCl concentrations on the lutein content of *Scenedesmus* sp. FSP3.

In the above experiments, *Scenedesmus* sp. FSP3 lutein content was indeed enhanced under light intensity stress and salt stress, but the extent of the enhancement was limited; the maximum lutein content obtained in the second stage of culturing under light and salt stress was 5.54 and 5.51 mg g^−1^ (Figure 3B,D), respectively. Therefore, it is necessary to optimize light stress and salt stress simultaneously, further stimulating *Scenedesmus* sp. FSP3 in order to accumulate lutein. Cultivation time also had an important effect on lutein, according to Figure 3D. The light intensity, NaCl concentration, and cultivation time were optimized by response surface methodology (RSM). The experimental design of the response surface methodology and experimental results are shown in Table S1. The response surface (Figure 4) and contour map (Figure S1) for lutein content were obtained by software analysis.

**Figure 5:**
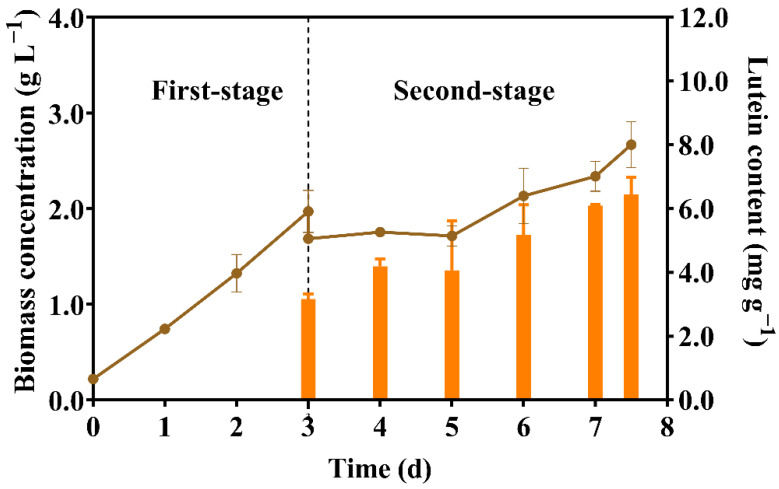
Growth and lutein content for *Scenedesmus* sp. FSP3 using two-stage culture under optimized conditions. In the first stage, *Scenedesmus* sp. FSP3 were cultured with added PC and 5% CO_2_, aerated for 3 days. For the second stage, they were cultured under a light intensity of 160 μmol m^−2^ s^−1^ and an NaCl concentration of 156.6 mmol L^−1^ for 4.5 days.

According to this model, the maximum lutein content was obtained when the cultivation time, light intensity, and NaCl concentration were 4.5 d, 160 μmol m^−2^ s^−1^, and 156.6 mmol L^−1^, respectively. Then, *Scenedesmus* sp. FSP3 was cultivated under these optimized conditions and the results of the growth and lutein content of *Scenedesmus* sp. FSP3 is shown in Figure 5.

The initial seeding density was 0.22 g L^−1^; after three days of culture, the biomass concentration reached 1.97 g L^−1^, and the maximum biomass productivity was 0.58 g L^−1^ d^−1^ (Figure 5). Then, we transferred the microalga cells to the second stage, where the growth of *Scenedesmus* sp. FSP3 was delayed for the following two days, then grew slowly. The lutein content of *Scenedesmus* sp. FSP3 was 3.17 mg g^−1^ at the beginning of the second stage but then gradually accumulated and reached a maximum of 6.45 mg g^−1^ after 7.5 days (Figure 5) under combined light and salt stress. The lutein productivity achieved was 2.30 mg L^−1^ d^−1^. By using this culture method, high lutein production and CO_2_ fixation were simultaneously achieved.

## 3. Discussion

*Scenedesmus* sp. FSP3 showed good biomass productivity and a good carbon fixation rate under CO_2_ concentration ranges of 5–20% (Table 1). The highest values for maximum biomass concentration (2.99 g L^−1^), maximum biomass productivity (0.41 g L^−1^ d^−1^), and maximum specific growth rate (0.77 g L^−1^ d^−1^) were all obtained at 5% CO_2_ after seven days of cultivation (Figure 1). In previous reports, *Chlorella sorokiniana* GS03 showed a maximum CO_2_ fixation rate of 0.66 g L^−1^ d^−1^ at 5% CO_2_ [14]. In our research, *Scenedesmus* sp. FSP3 also achieved a CO_2_ fixation rate of 0.77 g L^−1^ d^−1^ at 10% CO_2_. The maximum CO_2_ fixation rate of *Chlorella pyrenoidosa* SJTU-2 and *Scenedesmus obliquus* SJTU-3 was 0.26 and 0.29 g L^−1^ d^−1^ at 10% CO_2_, respectively [9]. The CO_2_ fixation rate of *Nanochloropsis* was 0.22 g L^−1^ d^−1^ under simulated flue gas (11% CO_2_, 10% O_2_, 1–2% CO, and 500 ppm CH_4_ and N_2_) [15]. The CO_2_ fixation rate of *Scenedesmus obliquus* PF3 reached 0.75 g L^−1^ d^−1^ within simulated flue gas (10% CO_2_ and 100 ppm NO) [16]. Referring to previous reports [14,17,18], *Scenedesmus* sp. FSP3 still performed well for CO_2_ sequestration under 15 and 20% CO_2_ concentrations (Table 1). In general, *Scenedesmus* sp. FSP3 had a relatively high CO_2_ fixation rate under a concentration of 5~20% CO_2_ and had the ability to rapidly convert CO_2_ into biomass.

In general, CO_2_ concentrations below 5% are suitable for microalgal growth [9], while higher CO_2_ concentrations are considered harmful to microalgal growth. CO_2_ toxicity is caused by the influx of H^+^ from the acidic extracellular space and intracellular H^+^ production through CO_2_ hydration [19]. The CO_2_ tolerance of different microalgae is species-specific, such as *Chlorella sorokiniana* UTEX 1602, which could tolerate 30% CO_2_ but grew best at 10% CO_2_, while *Chlorella vulgaris* ARC 1 grew best under a 6% CO_2_ concentration and was completely inhibited at 12% [20]. Besides, *Desmodesmus* sp. 3Dp86E-1-a, isolated by Solovchenko et al. [21], was reported to be able to grow at under 100% CO_2_.

When exposed to high CO_2_ levels, microalgae strive to maintain their cytosolic neutrality through CO_2_ tolerance mechanisms, such as (i) upregulating proton extrusion; (ii) inactivating the CCM; (iii) modifying the composition of the cell membrane to enhance its role as a proton barrier; and (iv) increasing ATP synthesis to provide bioenergy for the operation of ATP-driven transport proteins [19]. *Scenedesmus* sp. FSP3 had a high tolerance to CO_2_, growing well at under 30% CO_2_, which may reduce CO_2_ toxicity in this way. The higher biomass productivity and high CO_2_ fixation rate and CO_2_ tolerance of *Scenedesmus* sp. FSP3 makes it a great candidate for converting industrial gas CO_2_ into biomass.

CO_2_ solubilization enhancers facilitated high concentrations of CO_2_ to be more bioavailable to the microalgae, resulting in faster biomass productivity and CO_2_ fixation rate. It is reported that the CO_2_ solubilization enhancers can either increase the solubility of CO_2_ through their affinity with CO_2_ [11] or by increasing the driving force of the gas-liquid mass transfer process [12]. The *Scenedesmus* sp. FSP3 supplemented with PC showed better growth and carbon dioxide absorption. In a previous study, 0.5 g L^−1^ NaHCO_3_ was added to a culture of *Chlorella* sp. HS2 [10]; their biomass productivity reached 530 mg L^−1^ d^−1^, which is a 45% increase compared to that of the control. Zhu et al. [11] added 1 mM PEG 200 to the medium, and the specific growth rate of *Nannochloropsis oceanica* reached a maximum of 1.41 d^−1^, which was 21.5% higher than that obtained without PEG 200. Extensive research and the use of CO_2_ solubilization enhancers will help to further promote CO_2_ adsorption in tandem with microalgal bio-CCU [2].

Lutein, a photosynthetic auxiliary pigment, is closely associated with the light-harvesting complex (LHC) of photosynthetic apparatus, meaning the light intensity has a strong influence on it. Under low light conditions, lutein synthesis was enhanced in parallel with light-harvesting complex proteins to increase the amount of LHC and possibly capture low-incident light efficiently [13]. The same trend has been seen in previous studies; *Cocccomyxa onubensis* [22] was incubated at 50, 140 and 400 μmol m^−2^ s^−1^, with higher lutein content observed at 50 μmol m^−2^ s^−1^. *Chlorella sorokiniana* FZU60 [23] was grown (on day 4) under 150, 300, 450, 600, and 750 μmol m^−2^ s^−1^ light intensities, with the highest lutein content (9.81 mg g^−1^) produced under 150 μmol m^−2^ s^−1^ light intensity incubation, with the lowest (6.46 mg g^−1^) observed under 750 μmol m^−2^ s^−1^. When the light intensity reached the appropriate condition, the content of lutein decreased. This finding could be attributed to the increased light intensity, under which the photosynthetic apparatus is oversaturated with the breakdown of excess LHCs [8]. However, when the light intensity then continued to increase, lutein accumulated as a cellular defense against photooxidative damage to the photosynthetic system [24]; *Tetraselmis* sp. CTP4 [25] was reported to undergo the same condition, with higher lutein content obtained in the presence of a high light intensity of 170 μmol m^−2^ s^−1^ when compared to a low light intensity of 33 μmol m^−2^ s^−1^. Additionally, *Desmodesmus* sp. F51 [26] was grown at light intensities of 150, 300, 450, 600, and 750 μmol m^−2^ s^−1^ to assess the effect of light intensity on lutein accumulation, reaching a maximum lutein content of 5.05 mg g^−1^ at 600 μmol m^−2^ s^−1^. When light intensity continues to increase, it causes serious damage to the microalgal cells. The effect of light intensity on lutein accumulation is species-specific. This result suggests that optimizing the availability of light is critical for lutein accumulation in microalgal cells.

Excessive salinity (of the medium) is detrimental to the growth of microalgae. The high-salt-concentration hazards for microalgae include the following: water loss, ion imbalance, the overproduction of reactive oxygen species, the enzyme inactivation of cellular ions, and osmotic pressure imbalances [27]. Salt stress leads to several biochemical and bioenergetic changes, such as increased lipid synthesis and energy production, changes in membrane permeability due to disruption of ion homeostasis, and elevated levels of reactive oxygen species (ROS), resulting in changes in cellular metabolism as the microalgae adapt to their environment in response to stress and attempt to restore homeostasis in vivo [28], which leads to changes in the levels of antioxidant compounds that scavenge highly toxic ROS. Lutein, an antioxidant pigment, also accumulates in the cells of microalgae. Ali et al. [29] found elevated antioxidant activity in *Chlorella vulgaris* when 10 g L^−1^ NaCl was added, with a significant increase in total carotenoids, including lutein. Bermejo et al. [30] reported that *Coccomyxa onubensis* growth was inhibited when 200–500 mM salt was present, but lutein production was significantly induced by up to 7.80 mg g^−1^. The most suitable salt concentration for lutein accumulation is also species-specific. Excessive salt concentration affects microalgal cell activity in many aspects, thus affecting lutein accumulation, so finding the right salt concentration is also necessary for lutein production.

Through the combined action of light stress and salt stress, the content of lutein increased by 17.27% when compared to a single factor change; the combination of stress can further promote the accumulation of lutein in microalgal cells. Lutein productivity was higher than that reported in most of the related studies (Table 2), which was attributed to the rapid growth of microalgae during the first stage, which greatly shortened the incubation period.

By using a two-stage culture method, efficient CO_2_ fixation was achieved during the first stage, and high lutein production was achieved during the second. This proves that it is feasible to integrate lutein production and CO_2_ fixation processes in microalgae when using a two-stage culture strategy.

## 4. Materials and Methods

### 4.1. Microalgae Species and Cultivation

The microalgal strain used in this study was *Scenedesmus* sp. FSP3, which was provided by Jo-Shu Chang (National Cheng Kung University, Taiwan). BG-11 medium was used for the cultivation of *Scenedesmus* sp. FSP3. *Scenedesmus* sp. FSP3 culture was grown in a 0.5 L modified Erlenmeyer flask and maintained at 25 ± 1 °C, illuminated with 90 μmol m^−2^ s^−1^ fluorescent light, and 5% CO_2_ infusion at 0.1 vvm (volume gas per volume media per minute) flow rate, continuously (filtered through a 0.22 μm microporous filter). The light quantum meter was placed at the bottom of the incubator to measure the light intensity. After three days of cultivation, the culture solution was collected as an experimental seed. In the culture process, we used two reactors: an airlift reactor and a modified Erlenmeyer flask. The airlift reactor is basically a cylindrical glass bottle with a diameter of 5.5 cm and a height of 35 cm, with an effective volume of 0.5 L. There is a vent in the bottle that goes from the top to the bottom. The gas is aerated from the bottom to mix the microalgae. In the upper part, there is an opening for the addition and collection of the culture medium. In the second stage, the microalgae were cultivated in the 0.5 L modified Erlenmeyer flask with a diameter of 13 cm and a height of 16 cm. Ventilation and openings are the same as in airlift reactors. The reactors were incubated on racks with LED lights, which were illuminated from directly above. The intensity of the light can be adjusted by a knob.

### 4.2. Cultivate Model: Two-Stage Culture

*Scenedesmus* sp. FSP3 cells with an optical density (OD680 nm) of 0.5 were inoculated into BG-11 medium. In the first stage, *Scenedesmus* sp. FSP3 culture was grown in a 0.5 L airlift column photoreactor, illuminated with 90 μmol m^−2^ s^−1^ fluorescent light at 25 ± 1 °C. The optimal CO_2_ concentration for cell growth was assessed; *Scenedesmus* sp. FSP3 was cultivated with 0.03% (air), 5%, 10%, 15%, 20%, 25% and 30% CO_2_ concentrations, aerated at 0.1 vvm flow rate continuously, respectively. For better carbon sequestration, 5 mmol L^−1^ methanol, polyethylene glycol (PEG200), propylene carbonate (PC), polyethylene glycol dimethyl ether (NHD), and N-methylpyrrolidone (NMP) were selected as CO_2_ solubilization enhancers and were added to the microalgal culture. When the microalgae growth reached the end of the exponential phase, the microalgal cells were transferred to the second stage of the culture method. In the second stage, the *Scenedesmus* sp. FSP3 culture was grown in a 0.5 L modified Erlenmeyer flask and with air infusion rate of 0.2 vvm, and the microalgae were subjected to light and salt stress. For optimization of light intensity, the microalgae were illuminated with 30, 90, 150, 210 μmol m^−2^ s^−1^; for the optimization of the NaCl concentrations, the BG-11 medium was supplemented with 0, 50, 100, 150, and 200 mmol L^−1^ of NaCl. The dynamic changes in lutein content were measured under different light intensities and NaCl concentrations, respectively.

### 4.3. Measurement of Cell Growth

The biomass concentration of *Scenedesmus* sp. FSP3 was calculated by the optical density of *Scenedesmus* sp. FSP3 at 680 nm. The relationship between OD_680_ and the dry cell weight (DCW) of *Scenedesmus* sp. FSP3 was established by linear regression; the biomass concentration can be calculated by Equation (1).

(1) $$C=0.302x\;\;\;R^{2}=0.9981$$

where C is the biomass concentration (g L^−1^), and *x* is OD_680_.

Overall biomass productivity (*P_overall_*, g L^−1^ d^−1^) was calculated from the variation in biomass concentration (ΔC, g L^−1^) within a cultivation time (Δt, d), according to Equation (2):

(2) $$P_{Overall}=\frac{\Delta C}{\Delta t}$$

### 4.4. Measurement of Carbon Dioxide Biofixation Rate

The CO_2_ fixation rate, *R*_CO_2__ (g L^−1^d^−1^), was calculated using Equation (3):

(3) $$R_{{\mathrm{CO}}_{2}\;}=f_{C}P_{Overall}\left(\frac{M_{{\mathrm{CO}}_{2}\;}}{M_{C}}\right)=1.88\times P_{Overall}$$

where *f_C_* is the carbon content of the microalgal cells (%, *w/w*), and *M_C_* is the molecular weight of the carbon. *M*_CO_2__ is the molecular weight of the CO_2_. The value of *f_C_* becomes 0.51 by utilizing CH_1.83_O_0.48_N_0.11_P_0.01_ as the molecular formula for the microalgal biomass.

The input and output gas of the microalgae cultures were collected by a 0.5 L airbag; then, the CO_2_ concentration was measured by a CO_2_ analyzer. CO_2_ gas absorptivity (*η*_CO_2__) was calculated according to Equation (4):

(4) $$\eta_{{\mathrm{CO}}_{2}}=\frac{C_{{\mathrm{CO}}_{2}.in}-C_{{\mathrm{CO}}_{2}.out}}{C_{{\mathrm{CO}}_{2}.in}}$$

where *C*_CO_2__, in (%), is the CO_2_ volume fraction of the inlet gas, and *C*_CO_2__, out (%), is the CO_2_ volume fraction of the effluent gas [34].

### 4.5. Extraction and Determination of Lutein

The lutein was extracted from the *Scenedesmus* sp. FSP3 cells using a modified method by Xie et al. [26]. In brief, 10 mg of lyophilized cells and glass beads of 0.1 mm diameter were mixed with an aqueous potassium hydroxide solution (0.5 mL, 60% *w/w* KOH). The microalgal cells were crushed at 65 Hz for 10 min by a grinder. The mixture was then placed in a 40 °C water bath for 40 min. The lutein was extracted by ethyl ether, and the cell fragmentation and centrifugation were repeated four–five times. The supernatant was collected until the extract solution was colorless and the lutein was completely extracted. We evaporated all of the ethyl ethers in supernatants with nitrogen and added 3 mL of acetone to redissolve the residue.

After extraction, high-performance liquid chromatography (Agilent 1260 Infinity II, Waldbronn, Germany) was used to determine the lutein content. The binary-mobile phase consisted of (A) methanol-acetonitrile-water (80:10:10, *v/v/v*) and (B) methanol-acetonitrile (40:60, *v/v*) flowed through an Eclipse XDB-C18 column (4.6 mm × 150 mm × 5 μm) at 25 ℃ at 0.8 mL min^−1^. The lutein content was detected by measuring the absorbance at 447 nm.

### 4.6. Response Surface Methodology for Lutein Yield

The second stage of incubation involved the optimization of light intensity and salt concentration in promoting lutein production. Moreover, the cultivation time also had a significant effect on lutein production. Therefore, these three factors were selected: cultivation times of two, four, and six days, light intensity levels of 60, 110, 160 μmol m^−2^ s^−1^, and NaCl concentration levels of 100, 150, and 200 mmol L^−1^. The software Minitab 19 was used to design the Box–Behnken Design (BBD) for these three factors.

## 5. Conclusions

By using a two-stage culture method, the maximum biomass productivity (0.58 g L^−1^ d^−1^) and carbon fixation rate (1.09 g L^−1^ d^−1^) were achieved during the first stage; the addition of CO_2_ solubilization enhancers promoted the microalgae bio-CCUS. *Scenedesmus* sp. FSP3 reached a maximum lutein content of 6.45 mg g^−1^ and a lutein yield of 2.30 mg L^−1^ d^−1^ under the combination of light stress and salt stress during the second stage. This study integrates the lutein production process of microalgae with CO_2_ fixation, which improves overall economic efficiency and makes a positive contribution to the commercial production of lutein.

## Figures and Tables

**Table 1:** Comparison of maximum biomass productivity and CO_2_ fixation rates for different microalgae.

Microalgae Species	Gas Resource	Biomass Productivity (g L^−1^ d^−1^)	CO_2_ Fixation Rate (g L^−1^ d^−1^)	References
*Scenedesmus obliquus* PF3	10% CO_2_, 100 ppm NO	0.40	0.75	[16]
*Chlorella sorokiniana* GS03	5% CO_2_	0.35	0.66	[14]
*Heynigia riparia* SX01	15% CO_2_	0.38	0.71	[14]
*Scenedesmus dimorphus*	15% CO_2_, 400 ppm SO_2_, 300 ppm NO	0.35	0.66	[18]
*Scenedesmus obliquus* AS-6-1	20% CO_2_	0.15	0.29	[17]
*Scenedesmus obliquus* CNW-N	20% CO_2_	0.21	0.39	[17]
*Nannochloropsis* sp.	11% CO_2_, 10% O_2_, 1–2% CO, 500 ppm CH_4_, N_2_	0.12	0.22	[15]
*Chlorella pyrenoidosa* SJTU-2	10% CO_2_	0.14	0.26	[9]
*Scenedesmus obliquus* SJTU-3	10% CO_2_	0.15	0.29	[9]
*Scenedesmus* sp. FSP3	5% CO_2_	0.41	0.77	This study
	10% CO_2_	0.41	0.77	
	15% CO_2_	0.35	0.66	
	20% CO_2_	0.28	0.53	

**Table 2:** The lutein production of *Scenedesmus* sp. FSP3 compared with other microalgal strains under autotrophic conditions from previous reports.

Microalgae	Lutein Productivity (mg L^−1^ d^−1^)	Lutein Content (mg g^−1^)	References
*Chlorella sorokiniana*	0.84	3.00	[31]
*Coccomyxa onubensis*	0.55	6.20	[22]
*Chlorella minutissima*	0.67	6.37	[32]
*Scenedesmus almeriensis*	0.13	8.54	[33]
*Desmodesmus* sp. F51	0.65	5.50	[26]
*Scenedesmus* sp. FSP3	2.30	6.54	This study

## Data Availability

Data are available from the corresponding author: miaoxiaoling@sjtu.edu.cn.

## Supplementary Materials

The following supporting information can be downloaded at: https://www.mdpi.com/article/10.3390/molecules27217497/s1. Figure S1: Contour map of lutein content. (A) Contour map of different light intensities and cultivation time on lutein content, (B) Contour map of different NaCl concentrations and cultivation time on lutein content, (C) Contour map of different light intensities and NaCl concentrations on lutein content of *Scenedesmus* sp. FSP3; Table S1: Experimental design of response surface methodology and experimental results.

## Sample Availability

Samples from the compounds are available from the corresponding author: miaoxiaoling@sjtu.edu.cn.
