# Supplementary material for: A Two-Stage Culture Strategy for Scenedesmus sp. FSP3 for CO2 Fixation and the Simultaneous Production of Lutein under Light and Salt Stress
Source: Molecules. 2022 Nov 3;27(21):7497. doi: 10.3390/molecules27217497 (PMC9655217; doi:10.3390/molecules27217497)
Supplement: Supplementary file 1 [file molecules-27-07497-s001.zip › molecules-1935332-supplementary.pdf]

Table S1. Experimental design of response surface methodology and experimental results.

Number	Cultivation time (d)	Light intensity (μmol m^{-2} s^{-1})	NaCl concentration (mmol L^{-1})	Lutein content (mg g^{-1})
1	2	60	150	3.57
2	6	60	150	4.15
3	2	160	150	3.94
4	6	160	150	6.61
5	2	110	100	3.82
6	6	110	100	5.03
7	2	110	200	3.84
8	6	110	200	5.32
9	4	60	100	5.59
10	4	160	100	7.44
11	4	60	200	5.29
12	4	160	200	8.08
13	4	110	150	7.48
14	4	110	150	7.56
15	4	110	150	7.29

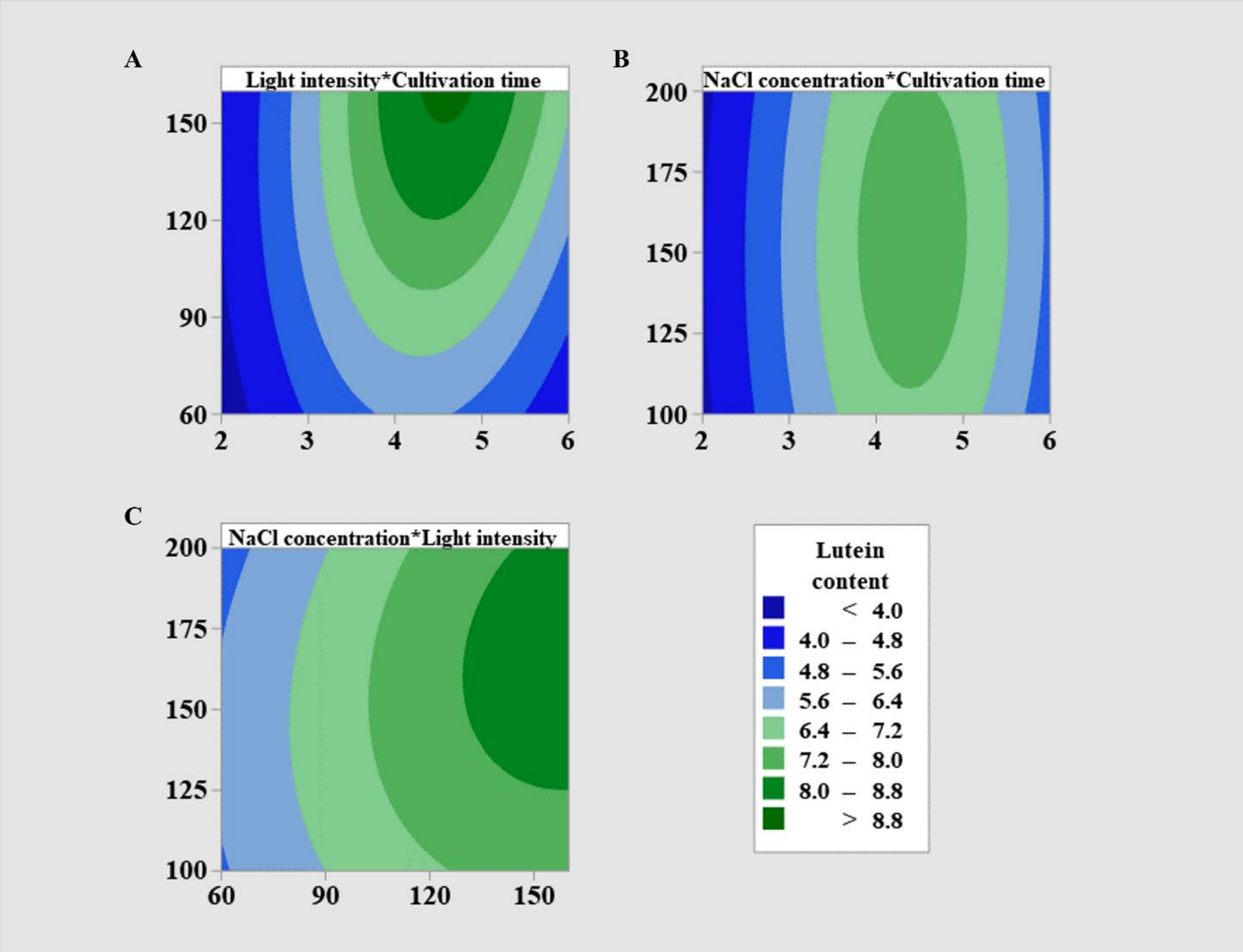

Figure S1. Contour map of lutein content. (A) Contour map of different light intensities and cultivation time on lutein content, (B) Contour map of different NaCl concentrations and cultivation time on lutein content, (C) Contour map of different light intensities and NaCl concentrations on lutein content of *Scenedesmus* sp. FSP-3.
